# Adding rituximab to chemotherapy for diffuse large B-cell lymphoma patients in Indonesia: a cost utility and budget impact analysis

**DOI:** 10.1186/s12913-022-07956-w

**Published:** 2022-04-25

**Authors:** Septiara Putri, Ery Setiawan, Siti Rizny F. Saldi, Levina Chandra Khoe, Euis Ratna Sari, Amila Megraini, Mardiati Nadjib, Sudigdo Sastroasmoro, Armansyah Armansyah

**Affiliations:** 1 Health Policy and Administration Department, Faculty of Public Health, University of Indonesia, Depok, West Java 16424 Indonesia; 2 Center for Health Economics and Policy Studies (CHEPS) University of Indonesia, Depok, West Java 16424 Indonesia; 3 Center for Clinical Epidemiology and Evidence Based Medicine (CEEBM) Cipto Mangunkusomo Hospital, Jakarta, 10430 Indonesia; 4 Community Medicine Department, Faculty of Medicine University of Indonesia, Jakarta, 10430 Indonesia; 5 Indonesian Health Technology Assessment Committee, Jakarta, 12950 Indonesia; 6 Center for Health Financing and Insurance, Ministry of Health Republic of Indonesia, Jakarta, 12950 Indonesia

**Keywords:** Rituximab, Lymphoma, DLBCL, Cost-effectiveness

## Abstract

**Background:**

Rituximab plus cyclophosphamide, doxorubicin, vincristine, and prednisone (R-CHOP) has been used to treat patients with diffuse large B-cell lymphoma (DLBCL) under National Health Insurance (NHI) scheme in Indonesia. This study aims to estimate its cost-effectiveness and budget impact.

**Methods:**

We conducted a cost utility analysis using Markov model over a lifetime horizon, from a societal perspective. Clinical evidence was derived from published clinical trials. Direct medical costs were gathered from hospital data. Direct non-medical costs, indirect costs, and utility data were primarily gathered by interviewing the patients. We applied 3% discount rate for both costs and effect. All monetary data are converted into USD (1 USD = IDR 14,000, 2019). Probabilistic sensitivity analysis was performed. In addition, from a payer perspective, budget impact analysis was estimated using price reduction scenarios.

**Results:**

The incremental cost-effectiveness ratio (ICER) of R-CHOP was USD 4674/LYG and 9280/QALY. If we refer to the threshold three times the GDP per capita (USD 11,538), R-CHOP could thus be determined as a cost-effective therapy. Its significant health benefit has contributed to the considerable ICER result. Although the R-CHOP has been considered a cost-effective intervention, the financial consequence of R-CHOP if remain in benefit package under National Health Insurance (NHI) system in Indonesia is considerably substantial, approximately USD 35.00 million with 75% price reduction scenario.

**Conclusions:**

As a favorable treatment for DLBCL, R-CHOP ensures value for money in Indonesia. Budget impact analysis provides results which can be used as further consideration for decision-makers in matters related to benefit packages.

**Supplementary Information:**

The online version contains supplementary material available at 10.1186/s12913-022-07956-w.

## Background

Non-Hodgkin’s lymphoma (NHL) has been identified as one of leading causes of cancer mortality [1, 2]. The most common type of non-Hodgkin’s lymphoma (NHL) is an aggressive tumor that affects B-lymphocytes; this is commonly known as diffuse large B-cell lymphoma (DLBCL), which accounts for approximately 30–40% of new cases of lymphoma [3]. DLBCL occurs primarily in adults or older patients; however, it could also occur in children [2, 4].

For decades, chemotherapy alone including cyclophosphamide, doxorubicin, vincristine, and prednisone (CHOP) has been the standard treatment for DLBCL patients, which provided favorable results regarding achievable partial or complete remission, as well as survival rates [5]. However, treatment and management of DLBCL patients significantly improved since the discovery of the targeted cancer drug that is rituximab (Rituxan, Mabthera), which is an anti-CD20 chimeric monoclonal antibody that targets normal and malignant B cells; this has demonstrated substantial clinical results when in combination with CHOP regimen [6–8].

Several clinical trials reported that adding rituximab and CHOP/CHOP like (R-CHOP) compared to CHOP alone as first-line therapy provided improvements particularly in terms of clinical response and outcomes among previously untreated DLBCL patients [9–14]. In young population with good prognosis, R-CHOP increased 3-year event-free survival (EFS) compared to CHOP (79% vs. 59%) as well as the overall survival (OS) (93% vs. 84%) [9]. Meanwhile, for elderly population aged ≥60 years, 3-year failure-free survival (FFS) provided promising evidence (53% for R-CHOP vs. 46% for CHOP patients) [11, 12]. The LNH 98.5 trial conducted by the Groupe d’Etudes des Lymphomes de I’Adulte (GELA) demonstrated that R-CHOP also showed its superiority for elderly group in terms of survival events. Regarding outcomes related to efficacy, event-free survival (EFS) for R-CHOP was 69%, while it was 49% for CHOP; moreover, the 12-month OS was 83% for R-CHOP patients, whereas it was 68% for CHOP patients. For the 2-year median follow-up, the EFS and OS were noted to be consistently higher than that in R-CHOP group [11, 12]. Furthermore, for the longer follow-up trial period, 10-year progression-free survival (PFS) was 36.5% vs. 20% and OS was 43.5% vs. 27.6% for R-CHOP and CHOP, respectively [14].

In Indonesia, epidemiological studies from 13 hematology centers reported that most Indonesian NHL patients were diagnosed with DLBCL (68.2%), followed by follicular lymphoma (10%), small cell lymphoma (5.3%), unclassified malignum lymphoma type (8%), and unclassified B cell type (2.3%).The numbers were noted to be slightly higher compared to DLBCL cases in Asian countries [15]. Rituximab, therefore, was recommended for all types of NHL; R-CHOP has been a standard for DLBCL patients, and it has been covered by the National Health Insurance (NHI) since 2014 [16]. Price per vial was considerably expensive, that is, about USD 214 (1 USD = IDR 14,000, 2019 value). Until 2017, according to Indonesian Health Security Agency/BPJS, the total claim of rituximab was reaching USD 10 million [17]. Hence, rituximab has ranked first among the top high claimed drugs in NHI system. Despite these favorable outcomes for DLBCL patients, there remains a need to further assess the cost-effectiveness of R-CHOP compared to CHOP in our setting to address concerns if it is of money’s worth.

Thus, the objective of this study was to conduct cost utility and budget impact analysis of R-CHOP and compare its results to that of CHOP for DLBCL patients. The result is expected to aid policymakers to consider existing benefit package decision in NHI program in Indonesia.

## Methods

### Target population

For this technology assessment, we conducted two types of data collection. First, clinical efficacy evidence was derived from published clinical trials. Second, costs and utility data were obtained primarily from hospitals and by interviewing patients. All these steps were conducted in accordance to the Indonesian Guideline on Health Technology Assessment [18].

The inclusion criteria are as follows: NHL patients with sub-type DLBCL who were assessed with standard diagnostic tests, those aged ≥18 years who were enrolled in the National Health Insurance program, and those who received R-CHOP as first-line therapy. Diagnosis confirmation procedures of DLBCL were done in accordance to the National Comprehensive Cancer Network (NCCN) [19] and Indonesian National Cancer Guideline [20]. All DLBCL patients were de novo and were characterized as untreated or have not received chemotherapy, radiation, surgery, or any targeted therapies prior to R-CHOP. In practice, rituximab is administered with a typical dose of 375 mg/m^2^ combined with CHOP injections. According to Indonesian National Formulary Guideline for NHI scheme, R-CHOP could be administered until eight cycles, with an interval of 3 weeks each cycle for DLBCL patients who were confirmed with mandatory immunophenotype CD20+ test [16]. Furthermore, majority of the patients were provided treatment in just 1 day, or had a short-term hospital stay as required. We have only collected data from patients who had completed at least the first three cycles of R-CHOP, to determine the changes in terms of the quality of life as well as magnitude of the costs.

### Model structure

**Fig. 1:**
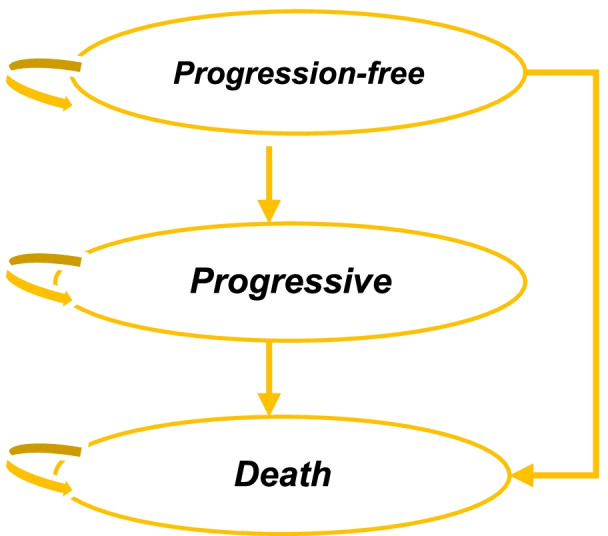
Markov Model

In order to assess the cost-effectiveness of R-CHOP as compared to that of CHOP for DLBCL patients, a cost utility analysis (CUA) was performed from a societal perspective. A model-based economic evaluation, that is, a Markov state transition model was, constructed to represent disease progression as well as prediction both for costs and outcomes. The model was conceptualized by reviewing previous published economic evaluation studies and also by combining clinicians and policymakers’ input that, in turn, represents the clinical practice in Indonesia. Lifetime horizon with 3-week cycle was applied. The Markov model structure is illustrated in Fig. 1. This mathematical model was run in Microsoft Excel 2016®.

Three mutually exclusive states were constructed, consisting of progression-free, disease progression (progressive), and death. Health states are defined by RECIL (Response Evaluation Criteria in Lymphoma) [21] and clinical judgement, which were then explored further from medical records in hospitals. In the initial state, patients were assumed to start in progression-free condition and remain on that state in a specific time, or move to progressive state or death. From progression disease state, patients either remain on the similar state or finally move to death. We assumed patients continuously progressed, without considering the relapse state. Chemotherapy types could change in progressive state with ICE (ifosfamide, carboplatin, etoposide) or DHAP (dexamethasone, high-dose ara-C, and Platinol) depending on specific medical conditions. However, very few patients received this chemotherapy compared to CHOP in Indonesia.

### Survival data and transition probability

Transitional probability is defined as the probability of patients’ movement from one state to another [22]. To generate transitional probability values, we explored the randomized clinical trials (RCTs) in systematic reviews that assessed the efficacy of adding rituximab to chemotherapy, specifically R-CHOP compared to CHOP. Each clinical trial study was assessed using Cochrane risk of bias and AMSTAR (A Measurement Tool to Assess Systematic Reviews) [23]. We therefore summarized data including overall survival (OS), progression-free survival (PFS), adverse events, tumor response, and relative risk (RR). Survival rates from relevant outcomes were transformed into probability that fitted to our model cycle. These probabilities values were then incorporated into an economic model, in addition to costs, outcomes, and mortality rate. (See formula 1 and 2)

1 $$r=\frac{-\mathit{\ln}\left(1-p\right)}{t}$$

2 $$p=1-{e}^{\left(- rt\right)}$$

Where p = transition probability; e = based on natural algorithm; r = rate; t = time.

### Resource use and costs

Societal perspective was used in this study; moreover, direct medical costs (DMC) and indirect costs (IC) were incorporated in the analysis. Mean costs were calculated by specified health states in the model. DMC included drug costs, visits/hospitalization, admission costs, laboratory, and diagnostic tests. We then recorded the data for each patient per visit to capture the treatment course. Cost data from 2015 to 2018 were collected retrospectively from hospital billing, and its value was adjusted in 2019 by applying Consumer Price Index (CPI) [22].

Direct non-medical costs (DNMC) and IC were primarily retrieved by interviewing patients and/or their caregiver(s) when they visited the hospital using structured questionnaire. Patients were prospectively included following the eligibility criteria for target populations. Informed consents were attached and explained to patients before direct interview. DNMC included transportation, meals (despite hospital/nutritional services), accommodation (since patients from other cities/provinces came to hospitals), or any spending outside hospital services. Moreover, IC including costs related to productivity loss due to hospital visit/stays were also estimated. Patient data were formally validated by an oncologist at each study site to ensure those met our inclusion criteria and fit the health states on economic model. A total of 60 patients included, 54 patients for progression free state and 6 patients for progressive state. Cost data finally specified into states in Markov model.

### Utilities

Similar with obtaining DNMC and IC information, data as regards quality of life were collected via direct interviews with patients using the standardized EQ-5D-5L instrument. This generic measurement instrument has five dimensions including mobility, self-care, usual activities, pain/discomfort, and anxiety/depression. Each dimension has five specific levels, that is, no problems, slight problems, moderate problems, severe problems, and extreme problems. Patients were also asked to complete the visual analogue scale (VAS) that was attached in the instrument [24]. There were 46 patients included, 41 patients in progression free state and 5 patients in progressive state. Furthermore, quality of life was translated into utility values by using Indonesian value set [25]. For this study, 3% discount rate was applied for both costs and benefits. All parameters mentioned above are presented in Table 1.

**Table 1:** Parameters included in economic model

Parameters	R-CHOP	CHOP	Distribution	Source
**Transition probabilities**^a^				
Progression free to death	0.004		Beta	WHO Life table
Progression free to progressive	0.370		Beta	GELA trial
Progressive to death	0.001		Beta	GELA trial
**Efficacy**				
RR Progression Free	0.55		Log normal	GELA trial
RR Progressive	0.53		Log normal	GELA trial
**Costs (in USD)**				
***Direct medical costs (DMC)*** ^b^				
Progression Free	431,4 ± 292,0	368,5 ± 389,0	Gamma	Hospital billing
Progressive	534,5 ± 134,0	368,5 ± 389,0	Gamma	Hospital billing
***Drug costs (DC)***				
Progression Free_Rituximab	643,0 ± 79,0		Gamma	Patient interviews
Progressive_Rituximab	563,4 ± 134,0		Gamma	Patient interviews
Progression Free_CHOP	50,7 ± 7,0		Gamma	Patient interviews
Progressive_CHOP	50,7 ± 7,0		Gamma	Patient interviews
***Direct non-medical costs (DNMC)*** ^c^				
Progression Free	86,8 ± 102,0	86,8 ± 102,0	Gamma	Patient interviews
Progressive	331,9 ± 328,0	331,9 ± 328,0	Gamma	Patient interviews
***Indirect costs (IC)***^d^				
Progression Free	144,2 ± 204,0	144,2 ± 204,0	Gamma	Patient interviews
Progressive	38,2 ± 19,0	38,2 ± 19,0	Gamma	Patient interviews
**Utility**				
Progression Free	0.74 ± 0.23		Gamma	Patient interviews
Progressive	0.48 ± 0.26		Gamma	Patient interviews
**Discount rate**				
Costs	3%		NA	HTA national guideline
Effect	3%		NA	HTA national guideline

^a^We adjusted and converted PFS and OS for transitional probabilities that fit into our model cycles and life time horizon (see supplemental material)

^b^direct medical costs assumed similar with progression free state

^c^direct non-medical costs are assumed similar with R-CHOP group

^d^indirect costs are assumed similar with R-CHOP group. In progressive state, very few patients use ICE with Rituximab

### Incremental cost-effectiveness ratio (ICER)

Health outcomes were represented as quality-adjusted life years (QALYs) and life years (LYs). QALY value was derived from multiplying length of life with utility values [22]. As a result of CUA, incremental cost-effectiveness ratio (ICER) as ratio of incremental costs and incremental effectiveness was calculated, in order to generate cost per QALY. We then generated ICER after implementing the discounts as well as half-cycle correction. The maximum threshold that we used is one to three times GDP per capita (1 GDP per capita = USD 3846), since Indonesia does not have country-specific threshold [18].

### Uncertainty analysis

Uncertainty potentially exist in economic evaluation studies, due to several factors such as methodological approach, model structure, input parameters, or mathematical assumptions which incorporated into cost-effectiveness model [26, 27]. Hence, to handle this, sensitivity analysis was applied. We performed probabilistic sensitivity analysis (PSA) by applying Monte Carlo simulations with 1000 iteration and estimate the impact simultaneously.

### Budget impact analysis

Budget impact analysis (BIA) was performed to assess financial consequences and evaluating the affordability when adopting the intervention [28]. With payer perspective, in this case BPJS, we projected several scenarios for 5 years. The assumption used were changes on drugs prices (reduction from 10% until 75%) and the scenario if implementing only chemotherapy as benefit packages for DLBCL patients. Parameter such as prevalence and new cases were calculated from BPJS’s claim data.

## Results

### Clinical evidence

In total, 471 journal articles were extracted from PubMed/MEDLINE, Cochrane Database of Systematic Review, and Center for Reviews and Discrimination (CRD) York (consisting of Database of Abstracts of Reviews of Effects (DARE), NHS Economic Evaluation Database (NHS EED), and Health Technology Assessment Database (HTA Database)) (as shown in Supplemental Material). Excluded were 29 duplicates and 342 irrelevant articles. Thus, in total, the remaining seven articles were used in this study for analysis [29–35].

Three articles by Zhang et al. [32], Hua et al. [34], and Fleury et al. [35] only reported the information about safety without clinical effectiveness such as survival rate of response rate. Then, the other four articles [29–31, 33], using systematic review/meta-analysis methods, revealed the safety and effectiveness of rituximab for DLBCL patients (see Supplemental Material). Finally, we found three articles which provided relevant trials to our study.

In total, three studies were found to have used randomized controlled trial methodologies to compare R-CHOP and CHOP for DLBCL patients with CD20+ [29, 30, 33]. From these three articles, the most relevant trials were chosen. The study from Coiffier et al. [11, 12] presented the first clinical trial of two regiment chemotherapies, that is, R-CHOP and CHOP. Then, Feugier et al. [13] and Coiffier et al. [14] revealed further clinical trial evidence from prior research, with Coiffier et al. [11, 12] having 5 and 10 years’ time observation (Table 2). In addition, the survival outcome from the three studies presented with EFS, PFS, and OS. Additionally, the tumor response rate and toxicity data from RCT were also illustrated in Supplemental Material.

**Table 2:** Survival evidence from published literature

Outcome	R-CHOP %survival (CI 95%)	CHOP %survival (CI 95%)	Relative risk (CI 95%)	***P*** value
**EFS**				
2 years	**57 (50–64)**	**38 (32–45)**	**0.55 (0.41–0.75)**	**< 0.001**
5 years	**47 (39.9–54.1)**	**29 (23.1–35.8)**	unreported	unreported
10 years	unreported	unreported	unreported	unreported
**PFS**				
2 years	unreported	unreported	unreported	unreported
5 years	**54 (46.8–61.6)**	**30 (24.4–37.3)**	unreported	unreported
10 years	**365 (29.7–43.5)**	**20.1 (14.6–26.2)**	unreported	unreported
**OS**				
2 years	**70 (63–77)**	**57 (50–64)**	**0.53 (0.37–0.77)**	**0.007**
5 years	**58 (50.8–64.5)**	**45 (39.1–53.3)**	unreported	unreported
10 years	**43.5 (36.4–50.4)**	**27.6 (21.4–34.3)**	unreported	unreported

*EFS* Event-free survival, *PFS* Progression-free survival, *OS* Overall survival

### Cost-effectiveness of R-CHOP

The economic model assumed that DLBCL patients with average age of ≥55 years receive R-CHOP or CHOP; this comes from the average age of patients from hospitals in Indonesia**.** Compared to CHOP alone, adding rituximab to CHOP shows significant benefit in LYG. The LYG for R-CHOP was 6.39 years, while it was 4.06 years for CHOP. In terms of QALY, the incremental QALY was 1.18, where RCHOP adding 4.18 QALY, and CHOP 3.00 QALY. From a societal perspective, the total lifetime costs for R-CHOP in DLCBCL patients were USD 105,847, while these amounted to USD 94,931 for CHOP (Table 3). The incremental costs between interventions were USD 10,916. The cost components such as drug costs and IC provided large portion in terms of calculating the total costs.

**Table 3:** Lifetime costs, life years gained (LYGs), quality-adjusted life years (QALYs), and incremental cost-effectiveness ratio (ICER)

Intervention	Costs (USD)	LYG	QALY
**R-CHOP**	105,847	6.39	4.06
**CHOP**	94,931	4.18	3.00
**ICER**		**4674/LYG**	**9280/QALY**

Costs are in USD (discounted)

The incremental cost-effectiveness ratio (ICER) of R-CHOP was USD 4674/LYG and 9280/QALY. If we refer to the threshold three times the GDP per capita (USD 11,538), R-CHOP is deemed potentially cost-effective. The significant health benefit contributed to the considerable ICER result.

**Fig. 2:**
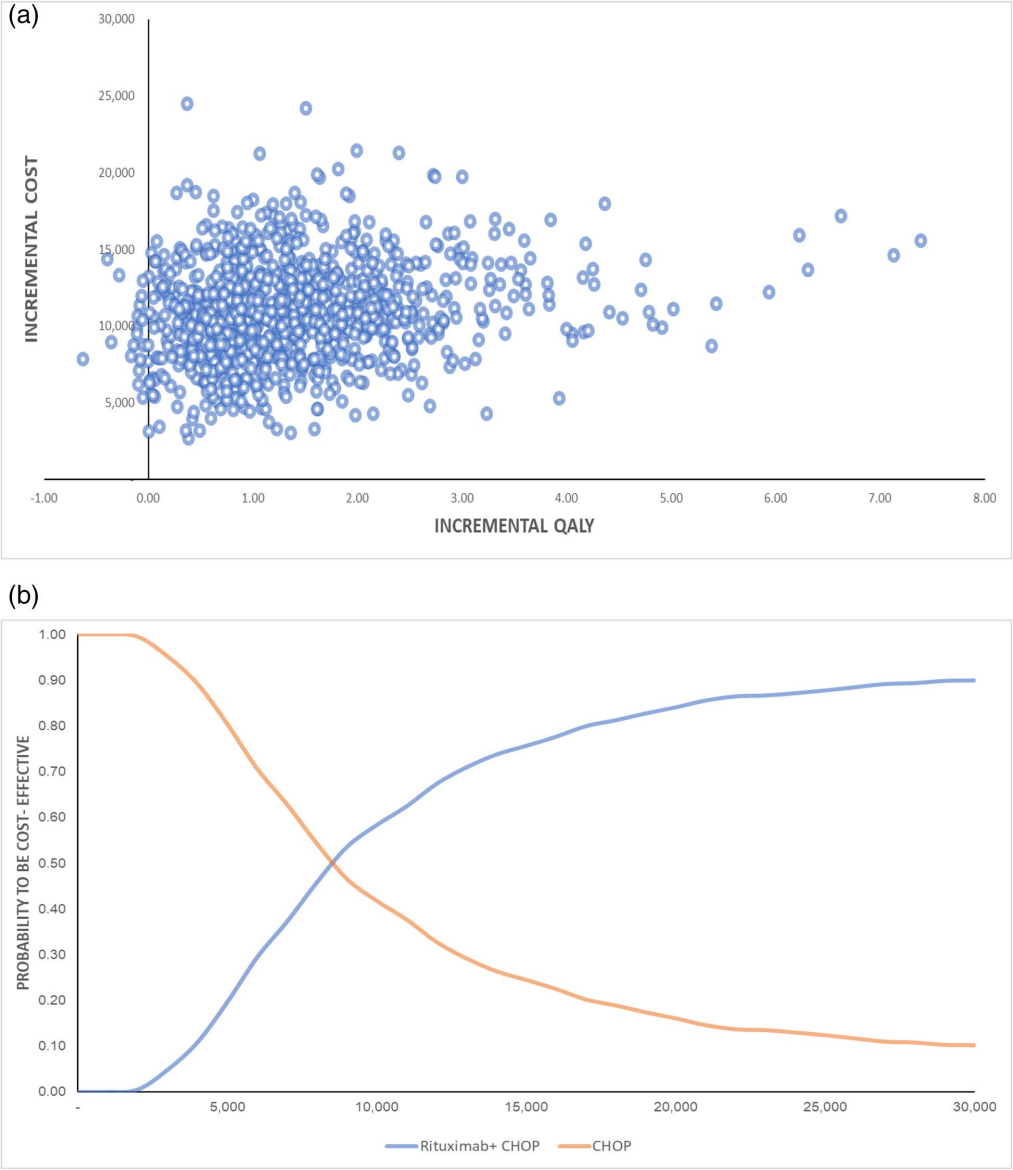
**a** ICE Scatterplot (**b**) Cost-effectiveness Acceptability Curve

The result of PSA is presented in Fig. 2, as illustrated by Incremental cost-effectiveness (CE) plot and cost-effectiveness acceptability curve (CEAC). The Incremental cost-effectiveness (ICE) scatterplot shows that as the incremental costs increased in accordance with the changes in incremental QALY, most values were scattered in 1–2 incremental QALY and incremental costs ranged USD 7200–15,000. Uncertainty existed, particularly for incremental QALY, which shows the extreme benefit of the therapy. At the maximum threshold per QALY gained (USD 11,538), the probability to be cost-effective for using RCHOP as first-line therapy for DLBCL was approximately 65%.

**Fig. 3:**
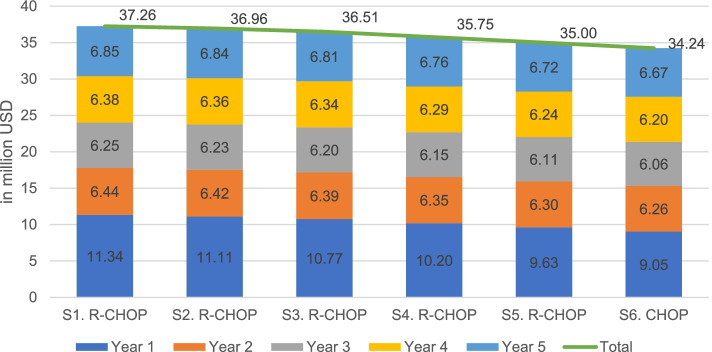
Budget Impact Analysis. S refers to “Scenario”. S1. R-CHOP = current price; S2 = reduced price by 10%; S3 = reduced price by 25%; S4 = reduced price by 50%; S5 = reduced price by 75%; S6 = CHOP only (1 USD = IDR 14,000)

Although the R-CHOP is deemed a cost-effective intervention, the result of this study followed by performing BIA to estimate budget in terms of payer affordability. By using assumptions with price reduction scenario, even with 75% price reduction, the total amount of budget was USD 35.00 million, it was slightly different with other 10, 25 and 50%, total budgets estimated were USD 36.96 million, USD 36.51 million and USD 35.75 million, respectively. Assuming that only CHOP was provided, the total budget would be USD 34.24 million. This, however, still has a substantial financial impact on NHI system, thus raising further discussions in terms of its affordability. The BIA result is presented in Fig. 3.

## Discussion

Our study indicated that combination of rituximab and CHOP for DLBCL patients in Indonesia setting is cost-effective, as proven by the favorable clinical outcome as well as economic consideration. This is aligned with published economic evaluation studies in several countries and settings.

Studies in European countries confirmed that R-CHOP has likely provided good value for money. Knight et al. [29] conducted model-based economic evaluation using UK health system perspective and reported that R-CHOP is considered as cost-effective therapy for both patients aged ≥60 years and < 60 years, with estimated ICER of £10,540/QALY and £7485/QALY, respectively. Similarly, cost-effectiveness study in Netherlands reported that compared to chemotherapy alone, R-CHOP provided significant QALY, that is, 0.88 for both younger and older population. The ICER is €13,983 and €17,933 per QALY gained, respectively [36].

Following the studies in Italy and France, R-CHOP provided promising result in terms of economic and health benefits. Utilizing the Italian health services perspective, Ferrara and Ravasion [37] reported that in young patients with good prognosis, adding rituximab to CHOP generated mean LYG 2.7 for R-CHOP and 2.5 years for CHOP. The mean costs per patient surprisingly are less expensive in R-CHOP, that is, €22,133 compared to CHOP (€22,831). Moreover, French-based study simulated economic evaluation within 15-year time horizon and estimated ICER for 12,259/QALY with significant incremental mean OS of 1.04 years [38].

In one academic hospital in Greece, R-CHOP was considered a favorable intervention for adult DLBCL patients, as it achieved 72% OS within 5 years. The ICER yielded €3394/LYG for R-CHOP and €1381/LYG for CHOP [39]. Meanwhile, in a study conducted in Canada, using population-based observational cohort, with 15-year time horizon, both for younger and older population, R-CHOP was found to be cost-effective. The ICER is US$19,144/QALY gained for younger population, and all ICERs per health outcomes were under US$10,000 [40]. Overall, economic evaluation studies both using model-based or local observational study identified R-CHOP as first-line treatment was cost-effective since the therapy has significant results in terms of survival and health outcomes in DLBCL patients.

This study has several limitations. First, due to the resources and time constraints, this study collected a limited number of samples for patients’ utility score, particularly those in a progressive state, which may influence the uncertainty on the economic model. Second, we did not specify the population by cancer staging since the hospital data were not fully available. We were therefore relying on model parametrization based on health states. Sub-group analysis might be useful to draw a more detailed economic evaluation result. Third, we did not categorize the patients as per their age, that is, young or older. The assumption used was average age that assumed close to elderly population, so the model might not fully represent a specific young population. Also, only people with good prognosis were incorporated into our model. Additionally, our model assumed that all patients complied with the treatment and chemotherapy cycles, while in reality that might be not true due to recorded dropouts or delayed visits to the hospitals. It obviously could influence the estimation of R-CHOP benefits.

In addition, although BIA predicted that the budget needed for 5 years was relatively high, there is no clear information about the ceiling standard in terms of affordability in the health system itself or compared to other technologies in NHI. High-level stakeholders’ discussion is thus needed for further discussion. Finally, our study demonstrated health economic evidence that could support the policymaking process on matters related to health benefit packages.

## Conclusions

Adding rituximab to chemotherapy (R-CHOP) is cost-effective for DLBCL patients in Indonesia compared to CHOP alone. Although the drug cost was relatively expensive, it remains to represent considerable value for money since R-CHOP provided significant health benefits. However, the financial consequence if rituximab remain in package is relatively high, and further consideration among decision-maker is required in terms of affordability standards. These findings are used to inform healthcare policy decision on benefit package on NHI scheme in Indonesia. Further studies including the sub-group analysis, exploring prognostic score, and adding study sites may be deemed favorable to support these current main results.

## Supplementary Information

**Additional file 1. **Clinical review.

**Additional file 2. **Costs.

**Additional file 3. **Utility.

**Additional file 4. **Transition probabilities.

## Data Availability

Data for costs and utility used in this study are available in supplementary material. More detail hospital-based data must contact and send the request to Indonesian HTA committee via kptk.online@gmail.com.

## Abbreviations

R-CHOP: Rituximab plus cyclophosphamide, doxorubicin, vincristine, and prednisone
BPJS: Badan penyelenggara jaminan sosial (Indonesian health security agency)
NHI: National health insurance
DLBCL: Diffuse large B cell lymphoma
DMC: Direct medical cost
DNMC: Direct non-medical cost
PFS: Progression-free survival
EFS: Event-free survival
OS: Overall survival
IC: Indirect cost
ICER: Incremental cost effectiveness ratio
LYG: Life years gained
QALY: Quality adjusted life years
