# Supplementary material for: Adding rituximab to chemotherapy for diffuse large B-cell lymphoma patients in Indonesia: a cost utility and budget impact analysis
Source: BMC Health Serv Res. 2022 Apr 25;22:553. doi: 10.1186/s12913-022-07956-w (PMC9040215; doi:10.1186/s12913-022-07956-w)
Supplement: Supplementary file 1 — Additional file 1. Clinical review. [file 12913_2022_7956_MOESM1_ESM.docx]

**SUPPLEMENTARY MATERIAL**

Supplementary material 1. Flow diagram of the review

Records excluded
(n = 342)

MEDLINE/PUBMED (n= 405)
Cochrane Database on Systematic Review (n= 7)

CRD York (n= 59)

## Identification

Full-text articles assessed for eligibility
(n = 100)

Records screened
(n = 442)

Records after duplicates removed
(n = 442 )

## Included

## Eligibility

## Screening

Studies included in qualitative synthesis
(n = 7)

Full-text articles excluded, with reasons
(n = 93 )

- Wrong patient population (n = 30)
- Wrong study design (n = 28)
- Not first line therapy (n = 6)
- Wrong indication (n = 4)
- Wrong intervention/outcomes (n = 4)
- Narrative review (n = 2)
- Non-english article (n = 1)
- Elderly population (n=1)
- Not full text (n =8)
- Not systematic review (n = 6)
- Not meta-analysis (n = 1)
- Mixed population without specific DLCBL data (n = 2)

Supplementary material 2. Flow diagram of the review

| ***Study name*** | ***Title*** | ***Population*** | ***Intervention*** | ***Comparator*** | ***Outcomes*** | ***Number of RCTs in SR*** | ***Number of Excluded RCTs in SR (reasons)*** | ***Number of Eligible RCTs in SR***  ***(Study ID)*** |
| --- | --- | --- | --- | --- | --- | --- | --- | --- |
| Meng et al., 2015 | Efficacy and safety of rituximab combined with chemotherapy in the treatment of diffuse large B-cell lymphoma: a meta-analysis | DLBCL | R-CHOP | CHOP | Complete response Overall response Overall survival Adverse effect | 10 | 7 (Chinese language)  1 (Chemo CEOP)  1 (escalated chemo) | 1 (Feugier 2005) |
| Fang et al., 2010 | A systematic review and meta-analysis of rituximab-based immunochemotherapy for subtypes of diffuse large B-cell lymphoma | DLBCL | R-Chemo | Identical chemo | Overall survival  Disease control Overall response | 6 | 2 (Chinese language)  1 (R-CHEOP)  2 (R-CHOP like)  1 (RTCOP) | 0 |
| Gao et al., 2010 | A systematic review and meta-analysis of immunochemotherapy with rituximab for B-cell non-Hodgkin’s lymphoma | B-cell non-Hodgkin’s lymphoma | R-Chemo | Identical chemo | Overall survival Progression-free survival Event-free survival Time to treatment failure Time to progression  Adverse events | 12 | 1 (R-CHOP like)  1 (Chinese language)  1 (R as maintenance)  8 (Mantle cell/follicular lymphoma/unspecified LNH) | 1 (Coiffier 2002) |
| Knight et al., 2004 | Rituximab (MabThera) for aggressive non-Hodgkin's lymphoma: systematic review and economic evaluation. | DLBCL | R-CHOP | CHOP | Event-free survival Overall survival Response rates Toxic effects | 1 | - | 1 (Coiffier 2002) |

Supplementary material 3. Quality Assessment

| **Quality assessment: AMSTAR** | **Meng 2015** | **Fang 2010** | **Gao 2010** | **Knight 2004** |
| --- | --- | --- | --- | --- |
| 1. Did the research questions and inclusion criteria for the review include the components of PICO? | Yes | Yes | Yes | Yes |
| 1. Did the report of the review contain an explicit statement that the review methods were established prior to the conduct of the review and did the report justify any significant deviations from the protocol? | No | Partial Yes | Partial Yes | No |
| 1. Did the review authors explain their selection of the study designs for inclusion in the review? | Yes | Yes | Yes | Yes |
| 1. Did the review authors use a comprehensive literature search strategy? | No | Partial Yes | Partial Yes | Yes |
| 1. Did the review authors perform study selection in duplicate? | No | Yes | Yes | No |
| 1. Did the review authors perform data extraction in duplicate? | No | No | Yes | Yes |
| 1. Did the review authors provide a list of excluded studies and justify the exclusions? | No | Yes | No | No |
| 1. Did the review authors describe the included studies in adequate detail? | No | Yes | Partial Yes | Partial Yes |
| 1. Did the review authors use a satisfactory technique for assessing the risk of bias (RoB) in individual studies that were included in the review? | No | No | Partial Yes | No |
| 1. Did the review authors report on the sources of funding for the studies included in the review? | No | Yes | Yes | No |
| 1. If meta-analysis was performed, did the review authors use appropriate methods for statistical combination of results? | No | Yes | Yes | No MA (only 1 study include) |
| 1. If meta-analysis was performed, did the review authors assess the potential impact of RoB in individual studies on the results of the meta-analysis or other evidence synthesis? | No | No | No | No MA (only 1 study include) |
| 1. Did the review authors account for RoB in individual studies when interpreting/discussing the results of the review? | No | No | No | Yes |
| 1. Did the review authors provide a satisfactory explanation for, and discussion of, any heterogeneity observed in the results of the review? | No | Yes | Yes | Yes |
| 1. If they performed quantitative synthesis did the review authors carry out an adequate investigation of publication bias (small study bias) and discuss its likely impact on the results of the review? | Yes | No | No |  |
| 1. Did the review authors report any potential sources of conflict of interest, including any funding they received for conducting the review? | Yes | No | Yes | Yes |
