# Supplementary material for: Adding rituximab to chemotherapy for diffuse large B-cell lymphoma patients in Indonesia: a cost utility and budget impact analysis
Source: BMC Health Serv Res. 2022 Apr 25;22:553. doi: 10.1186/s12913-022-07956-w (PMC9040215; doi:10.1186/s12913-022-07956-w)
Supplement: Supplementary file 2 — Additional file 2. Costs. [file 12913_2022_7956_MOESM2_ESM.pdf]

Costs (In Indonesia Rupiah)

1. **Direct Medical Costs (from hospital billing) R-CHOP**

		Progression Free			Progressive		
		**Rituximab**	**CHOP**	**Hospitalization**	**Rituximab**	**ICE***	**Hospitalization**
**Direct Medical Costs R-CHOP**	Mean	Rp10,594,889	Rp1,065,551	Rp5,667,423	Rp8,533,550	Rp4,066,422	Rp8,163,358
	Min	Rp1,529,000	Rp160	Rp109,440	Rp1,477,400	Rp1,621,603	Rp2,103,391
	Max	Rp57,419,876	Rp4,467,584	Rp33,010,258	Rp9,494,416	Rp14,652,275	Rp21,274,138
	Median	Rp9,523,405	Rp810,800	Rp4,548,646	Rp9,174,000	Rp2,024,781	Rp6,958,634
	StDev	Rp4,526,950	Rp646,925	Rp4,921,156	Rp2,238,700	Rp4,235,200	Rp3,993,546
	StError	Rp250,725	Rp36,108	Rp270,491	Rp674,994	Rp1,058,800	Rp998,386

In progressive states, CHOP costs assumed similar.

The use of ICE chemotherapy is very few (only 2 patients from 54 eligible patients), however we present the data to see difference compared to CHOP.

2. **Direct Non-Medical Costs**

		**Progression Free**	**Progressive**
**Direct Non- Medical Costs**	Mean	Rp1,225,236	Rp4,685,253
	Min	Rp70,000	Rp166,667
	Max	Rp5,839,333	Rp9,409,091
	Median	Rp733,333	Rp4,480,000
	StDev	Rp1,441,131	Rp4,624,629
	StError	Rp232,816	Rp969,590

3. Indirect Cost

By model states		Progression Free	Progressive
Indirect Costs	Mean	Rp 2,035,848	Rp 538,636
	Min	Rp 33,333	Rp 350,000
	Max	Rp 11,477,500	Rp 727,273
	Median	Rp 1,000,000	Rp 538,636
	StDev	Rp 2,877,552	Rp 266,772
	StError	Rp 713,596	Rp 154,021
