# Supplementary material for: Adding rituximab to chemotherapy for diffuse large B-cell lymphoma patients in Indonesia: a cost utility and budget impact analysis
Source: BMC Health Serv Res. 2022 Apr 25;22:553. doi: 10.1186/s12913-022-07956-w (PMC9040215; doi:10.1186/s12913-022-07956-w)
Supplement: Supplementary file 3 — Additional file 3. Utility. [file 12913_2022_7956_MOESM3_ESM.pdf]

Patient ID	Mobility	Self care	Usual activity	Pain	Anxiety	VAS	Value
P1	1	1	1	4	1	70	11141
P2	1	1	3	2	1	80	11321
P3	2	1	1	3	1	80	21131
P4	1	1	1	2	1	60	11121
P5	3	3	3	3	3	70	33333
P6	2	1	1	1	1	80	21111
P7	1	1	1	1	2	80	11112
P8	1	1	1	1	3	75	11113
P9	2	2	1	1	1	90	22111
P10	3	1	3	1	2	65	31312
P11	1	1	2	2	2	85	11222
P12	1	2	2	2	2	80	12222
P13	4	3	3	3	4	85	43334
P14	2	2	2	2	3	80	22223
P15	1	1	1	1	2	80	11112
P16	2	2	2	2	2	87	22222
P17	2	2	2	2	3	75	22223
P18	1	1	1	1	2	95	11112
P19	2	2	2	2	2	75	22222
P20	2	2	3	2	5	60	22325
P21	1	1	1	2	4	70	11124
P22	1	1	1	1	2	75	11112
P23	1	1	3	1	2	70	11312
P24	2	1	3	2	1	60	21321
P25	2	1	2	3	1	85	21231
P26	1	1	3	2	1	80	11321
P27	1	1	1	4	2	80	11142
P28	1	1	2	4	1	80	11241
P29	1	1	2	1	4	80	11214
P30	1	1	2	3	1	75	11231
P31	1	1	1	1	1	80	11111
P32	1	1	1	3	1	70	11131
P33	1	1	1	1	2	90	11112

P34	1	1	1	1	1	90	11111
P35	1	1	1	2	1	75	11121
P36	1	1	1	1	1	70	11111
P37	1	1	1	2	2	80	11122
P38	1	1	1	2	1	65	11121
P39	1	1	1	1	1	80	11111
P40	1	1	1	2	1	65	11121
P41	1	1	1	1	1	90	11111
P42	3	1	2	2	3	30	31223
