# Supplementary material for: Adding rituximab to chemotherapy for diffuse large B-cell lymphoma patients in Indonesia: a cost utility and budget impact analysis
Source: BMC Health Serv Res. 2022 Apr 25;22:553. doi: 10.1186/s12913-022-07956-w (PMC9040215; doi:10.1186/s12913-022-07956-w)
Supplement: Supplementary file 4 — Additional file 4. Transition probabilities. [file 12913_2022_7956_MOESM4_ESM.pdf]

Supplementary

Baseline Parameters showing transition probabilities from and to each health state

Transitions (from, to)	R-CHOP	CHOP	Distribution	Source
Progression free to progression free				
Interval 1	0.629	0.420	Beta	GELA trial
Interval 2	0.630	0.421	Beta	GELA trial
Interval 3	0.630	0.421	Beta	GELA trial
Interval 4	0.630	0.421	Beta	GELA trial
Interval 5	0.629	0.420	Beta	GELA trial
Progression free to death				
Interval 1	0.004	0.004	Beta	WHO life table
Interval 2	0.003	0.003	Beta	WHO life table
Interval 3	0.003	0.003	Beta	WHO life table
Interval 4	0.004	0.004	Beta	WHO life table
Interval 5	0.005	0.005	Beta	WHO life table
Progressive to death				
Interval 1	0.001	0.004	Beta	GELA trial
Interval 2	0.000	0.003	Beta	GELA trial
Interval 3	0.000	0.001	Beta	GELA trial
Interval 4	0.000	0.002	Beta	GELA trial
Interval 5	0.001	0.003	Beta	GELA trial

Note: All transitional probabilities were adjusted per 3 weeks. In transition probability from progression free to progression free and from progression to death for RCHOP, the time interval 1 equals to 0-24 months, time interval 2 equals to 24-48 months, time interval 3 equals to 48-72 months, time interval 4 equals to 72-96 months, and time interval 5 equals to 96-120 months. In transition probability from progression free to progression free and progressive to death for CHOP, the time interval 1 equals to 0-6 months, time interval 2 equals to 6-12 months, time interval 3 equals to 12-18 months, time interval 4 equals to 12-18 months, and time interval 5 equals to 12-18 months. In

transition probability from progression free to progressive for both RCHOP and CHOP, the time interval 1 equals to year 1, time interval 2 equals to year 2, time interval 3 equals to year 3, time interval 4 equals to year 4, and time interval 5 equals to year 5.
